# Case of Pericarditis, with Large Effusion, Marked by Cerebral Symptoms—Death

**Published:** 1866-03

**Authors:** Henry I. Bowditch


*Case of Pericarditis, with large effusion, marked by cerebral symptoms—death.* Read before the Boston Society for Medical Improvement, Dec. 26th, 1865, and communicated for the Boston Medical and Surgical Journal. By HENRY I. BOWDITCH, M. D.

A young man, aged 17, living in a healthy place in one of our suburban cities, was the patient. Born of a nervous family, his mother and brother had had chorea. He himself had never been very ill; never had rheumatism; had had a good appetite, and seemed perfectly healthy till actual attack.

Oct. 31, 1865.—I saw him in consultation, the case having been considered a very obscure one.

The history was as follows: Four weeks before he went to bed apparently in perfect health. He awoke during the night, chilly, but a hot foot-bath and warm drinks, &c., soon restored him, after slight vomiting of the sage tea he had taken for relief.

Next day, not feeling very well, he took salts and senna, and vomited anew; and then appeared pain in the left side of the thorax, with great oppression in breathing. The attending physician, on auscultation, found nothing marked about the physical phenomena of the heart or lungs. No distinct palpitations, no cough, no sputa. The next day there was a sudden attack of extreme orthopnœa, and the pulse was nearly absent for a few moments. Still, no marked physical phenomena on auscultation. On the contrary, by the account of the attending physician there was nothing manifest, even on a close examination.

On the following day all the thoracic symptoms suddenly yielded, and never after were prominent. The pulse recovered its character, the orthopnœa disappeared, and the patient was, at my visit three and a half weeks afterwards, able to lie down in any posture without apparent difficulty. A wholly new set of symptoms developed themselves, and were the only marked ones during that interval. Violent headache came on, with great flushing of the face and eyes, great restlessness, delirium, and strabismus, first of one eye and then of the other. For two or three days he was speechless. These cephalic symptoms for at least a week were quite severe, and were considered the only ones to be treated. After leeches and ice to the head, and active purges, and finally Bromide of Potassium, the more violent of them subsided, and when I saw him there was no flush of the face or strabismus; but there was, at times, a little wandering of mind at night, and nausea and great costiveness. He did not fully recover. One day his symptoms seemed to be typhoidal, in his dullness of intellect, some fever, &c., but the next he seemed brighter. His pulse was always rapid and feeble; his nights usually rather sleepless, with jactitation. The day before I saw him he had been quite drowsy. He had considerable nausea and vomiting a day or two previously, owing, however, apparently to medicine administered. The dejections had been normal, but rather infrequent and costive. Nothing remarkable noticed about renal excretion, but no special examination had been made.

I found him of rather small stature, with nothing striking in his aspect. He was lying on his back, quiet and rational, and without the least appearance of severe disease. His breathing was not at all hurried, and he spoke and moved in bed without any dyspnœa. His countenance was sufficiently bright; no strabismus. He answered promptly all questions, and with perfect intelligence, and during my whole examination he did not exhibit the slightest trace of cerebral disease. He did not look as much depressed or emaciated as I had anticipated finding him, after what had been said of his symptoms during the weeks preceding.

My impression, therefore, was that the cerebral symptoms that had occurred were not dependent on manifest organic changes, such as inflammation of, or effusion into the cavity of the cranium, but rather upon some sympathy with another part of the body.

Remembering the earlier and very transitory symptoms of pain in the chest, orthopnœa and pulseless condition, and the fact that at times Pericarditis is wholly lost sight of in the cephalic symptoms that occasionally accompany it, I looked to the region of the heart to see if an explanation could be found there.

On percussion I found dulness over the heart to three times the usual extent, viz: from the intercostal space between second and third ribs downwards, and in breadth corresponding. The impulse of the heart was scarcely, if at all, felt, and the sounds were very distant. A bellows murmur was heard high on the sternum, and down outside of the left nipple; not heard in the intervening space. The respiration was heard somewhat in both breasts, and in back throughout, without a trace of rales. The abdomen presented nothing of moment.

With these phenomena it was evident that pericardial effusion existed to a considerable amount. Deeming it probable that that was the primary and chief source of trouble, I suggested the application of ethereal tincture of iodine (ʒ ss. of iodine to ℥ i. of ether) outside, and one-fourth of a grain of digitalis three times daily, or *pro re nata.* With this a general tonic course of diet was indicated—milk and bread, chicken broth, &c. As under the Bromide of Potassium, given at night, the nervousness had somewhat lessened, I advised its continuance.

The pulse continued to fail, and the digitalis was omitted in forty-eight hours. I did not see him subsequently, but he failed rapidly, and died in six days afterwards, with the signs of increasing effusion; no return of cephalic symptoms.

At the autopsy Dr. Ellis found the pia mater at the base of the brain infiltrated, at some points, with pus, and the serum was more abundant than usual, and turbid. The brain itself presented nothing remarkable.

The pericardium was enormously distended by at least five pints of a purulent fluid, and large fibrinous masses. When the sternum and ribs were raised the pericardium was the only object visible, the lungs being wholly obscured by it. There were some patches of recent lymph over several parts of the heart, which otherwise was healthy. The other organs presented nothing remarkable.

The occurrence of nervous symptoms to such a degree, with pericarditis, is rare. Dr. Austin Flint has seen three. Two died undiscovered until after death.

The symptoms accompanying this state are peculiar; maniacal often. The patient may be speechless, as ours was, or he may spit in every direction, as in typho-mania. He at times seems frightened, and almost as if in delirium tremens. He may have convulsions, or coma. It is usual to find no marks of inflammation about the brain. In the present case it was slight, and evidently was not extensive enough to have caused death.

The prognosis in any case is generally not so much from the inevitable mortality of the affection, as from its usual entire latency; so that extensive effusion often takes place without being recognized.

Of course active leeching and blistering, or iodine over the heart, would be indicated in the early part of the disease, with a mild but sufficient diet, and subsequently stimulants, with wine and quinine, are of immense benefit. But at times paracentesis would really be called for. It is to be regretted that it was not tried in this case, as the enormous effusion, that rapidly increased after I saw him, would seem to have indicated its propriety. There could have been no objection to the operation, and scarcely any danger in tapping such a large sac as the pericardium became at last. The operation has been done by Schuh, of Vienna, Dessault, Beau, and others in France. Strictly speaking, there is no more danger in opening the pericardium than in tapping the abdomen, or the pleural, or any other serous cavity. All the usual arguments against it, such as that we might strike the heart, might wound the internal mammary artery, that we could not draw off the fibrin, that the advantage would be only temporary, as the fluid would re-accumulate, &c., would become no arguments against the operation, provided we were perfectly sure of our diagnosis of a *large* amount of fluid.—*Boston Medical & Surgical Journal.*

